# Addressing the Rising Trend in Early-Age-Onset Cancers in Canada

**DOI:** 10.3390/curroncol31070303

**Published:** 2024-07-19

**Authors:** Petra Wildgoose, Filomena Servidio-Italiano, Michael J. Raphael, Monika Slovinec D’Angelo, Cassandra Macaulay, Shaqil Kassam, Nancy Nixon, José Perea, Sarah Hamilton, Ravi Ramjeesingh, Sharlene Gill, Aaron Pollett, Shuji Ogino, Tomotaka Ugai, Abha Gupta

**Affiliations:** 1 Sunnybrook Health Sciences Centre, Toronto, ON M4N 3M5, Canada; michaelj.raphael@sunnybrook.ca; 2 Colorectal Cancer Resource & Action Network (CCRAN), Toronto, ON M4W 3E2, Canada; filomena.s@ccran.org (F.S.-I.); monika.s@ccran.org (M.S.D.); cassandra.m@ccran.org (C.M.); 3 Southlake Stronach Regional Cancer Centre, Newmarket, ON L3Y 2P9, Canada; skassam@southlakeregional.org; 4 Tom Baker Cancer Centre, Calgary, AB T2N 4N2, Canada; nancy.a.nixon@ahs.ca; 5 Department of Medicine, Institute of Biomedical Research of Salamanca (IBSAL), 37007 Salamanca, Spain; josepereag@hotmail.com; 6 BC Cancer Agency, Vancouver, BC V5Z 4E6, Canada; shamilton7@bccancer.bc.ca (S.H.); sgill@bccancer.bc.ca (S.G.); 7 Nova Scotia Cancer Centre, Dalhousie University, Halifax, NS B3H 1V8, Canada; ravi.ramjeesingh@nshealth.ca; 8 Division of Diagnostic Medical Genetics, Mount Sinai Hospital, Toronto, ON M5G 1X5, Canada; aaron.pollett@sinaihealthsystem.ca; 9 Department of Pathology, Brigham and Women’s Hospital, Harvard Medical School, Boston, MA 02115, USA; sogino@bwh.harvard.edu; 10 Department of Epidemiology, Harvard T.H. Chan School of Public Health, Harvard University, Boston, MA 02115, USA; tugai@hsph.harvard.edu; 11 Adolescent & Young Adult (AYA) Oncology Program, Princess Margaret Hospital, Toronto, ON M5G 2M9, Canada; abha.gupta@uhn.ca

**Keywords:** early age onset cancer, EAOC, EAOCRC, young onset cancer

## Abstract

A multi-disciplinary symposium on early-age onset cancer (EAOC) was held in October 2023 to explore challenges experienced by this rapidly growing population. A major outcome of the symposium was recognition of the remarkable similarities of EAOC patients’ journeys across cancer sites. Prevention and early detection of cancer are hindered by a lack of awareness among patients and family doctors that cancer can and does occur in younger persons. Distinct characteristics of the disease—such as a later stage at diagnosis and more aggressive tumor biology—require more potent treatments, which result in profound physical and psychosocial consequences that are unique to this age group. EAOC patient empowerment emerged as another key theme of the symposium. The development of a greater number of specialized clinics was called for, and patient support groups were recognized for the vital role they play in empowering patients and their families. Leading-edge medical advancements hold tremendous hope across the spectrum of EAOC care. New technologies based on genomic profiling, immunotherapy and microbiome alteration contribute to the development of highly effective, personalized approaches to treatment. All symposium participants expressed their commitment to speak with one resounding voice to advocate for equitable access to leading care practices for EAOC patients; thus, a fourth symposium is planned for November 2024.

## 1. Introduction

A symposium on early-age onset cancer (EAOC) was held in October 2023 to explore key issues affecting this rapidly growing population in Canada. This conference built on the outputs from two prior symposia on the subject of early-age-onset colorectal cancer (EAOCRC) which have been previously reported in this journal [1,2] by expanding the scope of participation to include all cancers affecting younger Canadians.

This symposium provided one of the first opportunities for healthcare providers, patients and advocacy groups representing various types of EAOC to come together to discuss their experiences. These discussions led to a recognition of several key themes and a call for collaborative action to address the needs of this unique patient population. A further symposium is planned in November 2024 to build on this work by developing specific strategies and recommendations directed to policy-makers and government.

### 1.1. Recent Trends in EAOC

**Figure 1:**
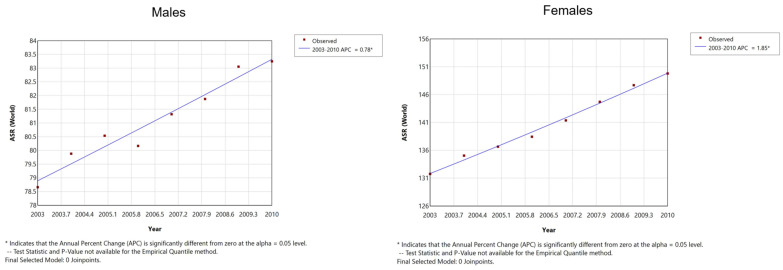
Age-standardized rate (ASR) of early-onset cancers in 44 countries combined, 2003–2010. Source: Dr. T. Ugai, personal correspondence.

Global trends show a significant increase in the incidences of many types of cancer in populations under 50 years of age, in stark contrast to declining rates of several types of cancer seen in older persons. These include cancers of the breast, colorectum, endometrium, esophagus, extrahepatic bile duct, gallbladder, head and neck, kidney, liver, bone marrow, pancreas, prostate, stomach, and thyroid [3,4]. This phenomenon is being seen across the developed world: recent studies have shown increasing incidences of cancers since the year 2000 among younger male and female populations in 44 developed countries, as shown in Figure 1.

In Canada, as in many other developed countries, EAOC is still relatively uncommon, accounting for less than 10% of all cancers diagnosed [5]. However, taken together across disease sites, the rapidly rising incidence of all EAOC populations represents a significant emerging clinical concern.

The increasing incidence of CRC and other GI cancers in younger patients had been identified earlier than other disease sites [6] and has therefore been more widely recognized. However, the surge in EAO cancers of many other types has now become a widespread trend [7]. For example, rates of metastatic breast cancer in women under 40 in the United States have risen 3.5 percent each year between 2004 and 2017 [8].

Studies over the past two decades have revealed common aspects across EAOC disease sites which point to potential improvements in prevention and care of this unique population. Many EAOCs share the same risk factors [3], are diagnosed at later stages of disease than those of their older counterparts [9], and their tumors show more lethal characteristics [9,10]. For example, several GI cancers share the risk factors of obesity, diet, lifestyle, environmental exposures and microbiome characteristics [9]. Younger women diagnosed with breast cancer are more likely to experience a recurrence at 5 and 10 years after therapy compared to older women [8].

### 1.2. Symposium Goals

The goals of this symposium were:

- To explore and promote awareness of the experiences of younger patients across the cancer continuum;
- To learn from leading practices in translating new research findings into practice;
- To capture emergent themes concerning common challenges faced by EAOC patients to guide future discussion and to consider as a basis for collaborative action in a future symposium.

Details of the symposium organization and agenda can be found in Appendix A.

## 2. Challenges throughout the EAO Cancer Patient Journey

Younger Canadians experience an array of challenges throughout their cancer journeys that are divergent from those of their older counterparts. The previous two symposia on EAOCRC discussed these in detail as they relate to colorectal cancer [1,2]. A resounding theme of the present symposium was the similarity of experiences, including clinical and psychosocial care, among EAOC patients across disease sites.

### 2.1. Identifying Risk Factors

The steady rise in the incidences of many types of cancer in persons born after 1950 suggests that their etiology may somewhat differ in important ways from that in older persons.

The study of the environmental and genetic causes of EAOC is challenging for several reasons. The interplay among environmental exposures, hereditary predispositions, the microbiome, and co-existing chronic diseases is complex; establishing cause-and-effect relationships is therefore difficult [3]. The growing but still relatively small size of the EAOC population requires decades of follow-up in prospective epidemiological studies. In addition, because genomic testing is not conducted routinely in the EAOC population, databases are incomplete. Finally, it is difficult to obtain accurate information about an individual’s past behaviours and exposures when a cancer diagnosis is made decades later. Scientists at the symposium called for new interdisciplinary research frameworks to study rare, complex diseases including EAOC and other illnesses. Large, prospective, life-course studies could identify prevention measures for EAOC concomitantly with other diseases [8,11].

Although in its early stages, actionable progress has been made in identifying risk factors for EAOC. For example, approximately one in six cancer patients is known to have a hereditary mutation associated with their cancer [11,12,13,14]. Patients who know their hereditary genetic profile can seek screening at an earlier age and can advise family members of their potential risk for developing cancer. Although testing for pathogenic germline genetic variants is widely available in Canada, there are gaps in access across jurisdictions and with respect to how many mutations are tested for.

In addition, research is underway to determine the impacts of dietary, lifestyle and environmental risk factors on the development of EAOC. Trends such as increasing obesity, type 2 diabetes, physical inactivity, Western-style diet, sugar-sweetened beverage intake, alcohol consumption, smoking, sleep patterns, reproductive factors and exposures to ionizing radiation and chemical carcinogens are being investigated with respect to EAOC [3,15,16].

### 2.2. Delayed Detection and Diagnosis

Delayed detection and diagnosis is a hallmark of EAOC and is consistent across disease sites. Patient participants at the symposium consistently reported long wait times for consultation with their primary healthcare provider and multiple referrals prior to a diagnosis. Published studies have reinforced this experience. Compared to people over 50 years of age, younger CRC patients presenting with symptoms experience longer delays to diagnosis [17,18,19], a factor that has been shown to contribute to a later stage of disease at diagnosis [20,21,22,23] and has been linked to poorer outcomes in certain populations [24,25,26,27].

Lack of awareness of cancer risk factors, signs and symptoms among younger Canadians is a particularly important issue. Patients who participated in the symposium stated that their diagnosis had been unexpected; most had been unaware of their risk profile and of the symptoms of cancer, and this was unanimous across disease sites.

Lack of awareness by primary healthcare providers of the possibility of cancer was also repeatedly emphasized by patient participants. Part of the reason for this is that EAOC, although rising rapidly, represents less than 10% of cancers diagnosed in Canada [5] and is seen uncommonly in family practice. Family history of cancer is an important risk factor that is often overlooked by primary care providers [18,28]. Additionally, symptoms of various types of EAOC often appear benign and open to interpretation [29]. Because the healthcare system’s approach to diagnosis is probabilistic, health professionals reported that their bias is “You’re too young to have cancer”.

Early detection and diagnosis is largely dependent upon access to primary care. Younger Canadians are particularly affected by the rising shortages of primary care professionals. Nearly one in three Canadians aged 18–34 is not registered with a family doctor [30] compared to one in five Canadians in general [31]. They may therefore be unable to obtain routine pelvic, breast or general physical examination, or to be questioned about their family history of cancer.

Younger patients who have a family history of cancer are deemed high risk and qualify for inclusion in an organized breast or colorectal (CRC) screening program where they are automatically invited to be tested. Such a referral, however, must be made by a physician for someone under age 50. (Of note, in the same month as the symposium was held, the Canadian province of Ontario announced that its breast cancer screening program allow self-referral of women aged 40 and above.)

### 2.3. Clinical Care Impacts

Once diagnosed, younger cancer patients in clinical care settings experience unique physical and psychosocial impacts [32]. Symposium participants noted that, in comparison with their older counterparts, disparities exist due to the greater severity of their disease, differences in their tumor molecular profiles, and their earlier stage of life. The experiences of EAOC patients across disease sites were remarkably similar.

Researchers and clinicians reported that the physical impacts of an EAOC diagnosis relate to the severity of disease and its treatment, as has been cited in the medical literature. EAO tumors are usually more advanced and more aggressive than those seen in older patients [20,21,22,23,24] and treatment regimens often have greater immediate and long-term effects as a result. Treatments affect patients in ways that are unique to an EAOC population, for example by impacting sexual health, body image and fertility. Concerns about fertility preservation were cited by patient participants as being paramount, reinforcing the conclusions of published studies [33,34,35]. Fatigue, although common for both EAOC and older cancer patients during and after treatment [36], was mentioned frequently by symposium participants as being particularly debilitating for younger patients who are simultaneously managing busy family lives and careers.

At the system level, participants in the symposium described gaps between jurisdictions and institutions across Canada in the availability of medical advancements, resulting in inequity of care and consequently of patient outcomes. For example, tumor profiling can potentially allow for more direct and less invasive treatment options to be considered; however, not all treatment centers offer these tests. Instead, patients receive standard-of-care therapeutic regimens that are based upon empirical evidence in older patients whose tumor profiles can differ significantly from those of EAOC patients.

For EAOC patients, the unique physical and psychosocial consequences of cancer and its treatment are intertwined and profound, with wide-reaching implications. Because EAOC has only recently been the focus of research investigations, there is not yet a full understanding of how these psychosocial impacts affect not only the patient but also their families, the healthcare system and society, in both the short and long term.

Patients representing many disease sites repeatedly raised concerns about taking time off work and finding childcare while undergoing treatment. Due to their earlier life-stage, younger Canadians with cancer often struggle to meet their financial obligations without the backstop of savings or government-supported programs for seniors to cover their loss of earnings and to pay for treatments not covered by the public healthcare system, such as oral cancer drugs (as has also been described in the literature [37]). In addition, during the survivorship phase, patients reported that they often struggle with finding their place within society and return-to-work [38].

Psychologically, EAOC patient panelists consistently reported intense feelings of isolation and loneliness because both their peers and the older patients they encounter have little in common with their disease-related experiences. Often, EAOC patients experience guilt and self-blame upon receiving a diagnosis of cancer. Anxiety and depression are known to be greater among EAOC patients than in their older counterparts [39,40].

## 3. Meeting the Challenges through Patient Empowerment

EAOC patients and clinicians participating in the symposium expressed that now more than ever this age cohort desires to be part of the solution to these challenges. Because obstacles to care are now appearing with greater frequency and with considerable deleterious effects, young patients who are technologically adept and information-hungry are emboldened to be at the forefront of change. Empowering patients to be self-advocates can bolster confidence at a time when they feel a loss of control as a result of their diagnosis. The involvement of EAOC patients can also help to ensure that their oncology teams are providing optimal care at a time when practitioners are still learning about how to manage these cancers of increasing incidence.

### 3.1. Empowering EAOC Patients through Individualized Care

Symposium participants repeatedly emphasized the need for a personalized, patient-centered approach to EAOC care encompassing primary care, specialized care and patient support groups. This method focuses evidence-based treatments and services on meaningful, patient-defined objectives.

Designing a program of individualized care for EAOC patients requires the input of all stakeholders, but particularly the patient. Several key elements must be in place:

- Patients must be partners in key clinical decisions, for example potentially delaying urgent cancer treatment until fertility preservation consultation is completed.
- Information exchange must occur effectively and expeditiously throughout the cancer care continuum so that EAOC patients feel fully informed about their disease, treatment options, and how their daily lives and future health will be impacted. Reputable and responsive online sources of data coupled with thoughtful and engaging conversations with healthcare providers are therefore essential. EAOC patients also described a desire to be aware of medical advancements and clinical trials that may offer them benefit.
- Ongoing support is needed to assist with healthcare system navigation, beginning with the patient’s first encounter and at key points throughout their care pathway. Because younger Canadians have had fewer interactions with the health care system, navigating the complexities of an unfamiliar system can be overwhelming, particularly after receiving a sudden and unexpected diagnosis.
- The scope of available services must address mental health and psychosocial factors, such as disruptions to patients’ familial responsibilities, careers, finances and social environment, by providing emotional and practical supports.
- Treatment options should be based on evidence from younger populations and not simply an extension of existing practices.

### 3.2. Specialized Clinics for EAOC Patients

The concept of individualized care is exemplified by specialized clinics that have recently been established to address the growing population of young adults with cancer. Two examples of leading practices were presented at the symposium:

- The Odette Cancer Centre in Toronto, Canada offers a holistic, individualized approach to younger patients. Originally established for CRC patients, it has recently expanded to include other types of cancer.
- Also in Toronto, the Princess Margaret Cancer Centre program for adolescent and young adult (AYA) cancer patients consists of a virtual team anchored by full-time positions of Advanced Practice Nurse and Program Coordinators.

These clinics operate virtually, promoting healthcare equity to patients living at a distance and reducing the stress of having to take time away from careers, school, or family to attend in person. Because they function within healthcare institutions, the programs use pre-existing resources. Individualized cancer management pathways are developed for each patient and comprehensive care is provided through a coordinated team involving oncologists, allied health professionals, and patient support groups. Patient resources are available on the programs’ websites. Social isolation is addressed through virtual group activities or peer-to-peer support. Based on the success of these programs, outreach to regional cancer centers has resulted in similar programs being initiated by these institutions.

### 3.3. Empowering Patients through Peer Organizations

Patient groups play an invaluable role in supporting younger Canadians diagnosed with cancer. They provide reputable sources of information about the disease, its treatments and medical advancements; advocate for patients’ needs; and build mutually beneficial relationships with healthcare professionals. At the population level, patient support groups actively promote prevention and early detection of cancer through outreach programs.

Cancer patient groups are also respected partners within the healthcare system. They bring together patients, healthcare professionals, researchers, policy-makers and legislators to focus on salient issues and advocate for advancements which promote the patient voice in decision-making.

## 4. Meeting the Challenges of EAOC through Medical Advancements

Experts offered examples of medical advancements in early-age onset colorectal cancer (EAOCRC). (EAOCRC was used as an example because this disease site is well researched compared to other disease sites.) The goal of these sessions was to share information on advancements in colorectal cancer research and care that may be generally applicable to other disease sites and which may promote timely and equitable access across Canada. Five areas of medical advancements were discussed at the symposium: genomic testing, immunotherapies, the microbiome, surgical treatments, and decentralized clinical trials. (A detailed summary of these advancements can be found in Appendix B).

Medical advancements are particularly important to EAOC patients. They allow for a more individualized approach to care which can better meet the unique needs of this patient population and for improved management of advanced/metastatic disease, which is more common in the younger population. An individualized approach is essential because standard treatment regimens are based on evidence gained in older populations which is often not relevant to younger patients. In addition, the evolution of tailored treatments is having profound impacts on younger patients and their families through improved side-effect profiles and survival, enabling younger patients to continue leading their busy lives. For example, precision risk stratification through genomic testing enables clinicians to identify mutations that respond to targeted therapies, so that treatment regimens can be tailored to reduce side-effects and improve outcomes and quality of life. Another example is the decentralization of clinical trials which can promote equitable access to and participation in studies by EAOC patients across the country, while also allowing for a more individualized approach to patient care.

Timely and equitable access to newly approved advancements in EAO cancer care is crucial for young cancer patients; however, their availability is unfortunately fragmented across Canada. Bringing together clinicians, patients, researchers and policy-makers to discuss these disparities was an important first step in identifying the challenges and beginning conversations about how leading practices could be adapted across jurisdictions. This symposium shared information on medical advancements that have proven successful in EAOCRC care. Advancements in care for other EAO cancer sites were not included in discussions at this symposium. The next step is to explore these developments, and their possible application to other disease sites through multi-disciplinary discussion at the next EAOC symposium, planned for November 2024.

## 5. Conclusions

This two-day symposium covered a broad range of cross-cutting issues across disease sites, as defined by EAOC patients, support groups, healthcare providers, researchers and policy-makers from across Canada and around the world. As the symposium progressed, the discussions brought to light several common themes and concluded with a strong call for cross-disciplinary action to advocate for change. Although the meeting organizers had originally intended to host only three symposia in this series, the high level of interest in pursuing the themes that emerged from the present meeting prompted the planning of a 4th symposium in November 2024. This next symposium will be focused on further collaborative development of the common themes outlined below into a concerted plan of action.

### Key Themes for Collaborative Action and Change

Several key themes were identified for collaborative action:

1. The unique needs of adults with EAOC should be addressed by establishing additional young adult cancer clinics throughout Canada. Leading examples of effective approaches can be adapted by cancer centers across the country, using their existing resources and facilitated by communications technologies.
2. Lack of awareness of cancer risk factors, signs and symptoms among younger Canadians and primary healthcare providers remains a major obstacle to the prevention and early detection of EAOC across several disease sites. Education and awareness campaigns are needed to increase the index of suspicion of cancer for primary care providers and to inform younger Canadians about the risks of cancer in their age group.
3. Re-evaluation of screening programs is needed to capture a younger population that is increasingly at risk for several types of cancer. While some Canadian provinces have recently taken action to reduce the screening age for breast cancer (Ontario, Alberta), these standards need to be applied across the country. Pan-Canadian screening guidelines for CRC should be reassessed to consider lowering the age limit to 45, as was achieved recently in the United States. Screening strategies for lung cancer should be evaluated against the needs of younger patients (most of whom are non-smokers) to ensure that their symptoms are not dismissed. Cervical cancer screening programs, which already include younger populations, should include HPV testing as a further safeguard.
4. Investment in the development of tests and therapies directed at key biomarkers in younger cancer patients is needed. Further research into genomic profiling will inform strategies for risk stratification, screening, diagnosis and treatment at the individual and population levels. Synchronized regulatory approvals for targeted therapies and their companion biomarker tests are needed to ensure timely access.
5. The potential of the expanding array of biomarker tests should be optimized by creating a national strategy to ensure uniform quality standards and equitable and timely access. In the immediate term, genomic profiling should be incorporated into all patients’ cancer care plans.
6. Support is needed for new interdisciplinary research frameworks to study rare, complex diseases, including EAOC and other illnesses.
7. Successful new techniques in the treatment of EAOCRC should be examined for potential benefit in other types of EAOC.

Through one resounding voice, all partners can work together to address the rising trend in EAOC.

## Data Availability

The data presented in this report are available in this article.

## Appendix A. Symposium Organization and Agenda

### Appendix A.1. Symposium Organization

The symposium was organized under the auspices of the Colorectal Cancer Resource & Action Network (CCRAN), which provides CRC patient and caregiver support, education and advocacy in Canada. It was co-chaired by Dr. Petra Wildgoose, Dr. Michael Raphael and Dr. Sharlene Gill and was moderated by Dr. Monika Slovinec D’Angelo.

An Expert Steering Committee provided direction on the symposium objectives, agenda, speakers and invited participants. Committee members were:

- Dr. Michael Raphael (Odette Cancer Centre, Sunnybrook Health Sciences Centre, Toronto, ON, Canada)—Co-Chair;
- Dr. Petra Wildgoose (Young Adult Colorectal Cancer Program, Sunnybrook Health Sciences Centre, Toronto, ON, Canada)—Co-Chair;
- Dr. Sharlene Gill (Division of Medical Oncology, University of British Columbia, Vancouver, BC, Canada)—Co-Chair;
- Dr. Darren Brenner (Departments of Oncology and Community Health Sciences, University of Calgary, Calgary, AB, Canada);
- Dr. Mary De Vera (Department of Pharmaceutical Sciences, University of British Columbia, Vancouver, BC, Canada);
- Dr. Clarence Wong (Division of Gastroenterology, University of Alberta, Edmonton, AB, Canada);
- Dr. Daniel Schiller (Department of Surgery, University of Alberta, Edmonton, AB, Canada);
- Dr. Mary Jane Esplen (Department of Psychiatry, University of Toronto, Toronto, ON, Canada); and
- Dr. Christopher Lieu (Division of Medical Oncology, University of Colorado, Boulder, CO, USA).

### Appendix A.2. Participation

Registrants included 347 health care professionals, patients and caregivers, and healthcare policy-makers. Registrants were from Canada, the United States, Great Britain, Ireland, Australia, Denmark, Netherlands, Bulgaria, Pakistan, India, Dominican Republic, Egypt, South Africa, Ghana, Portugal and Japan.

### Appendix A.3. Agenda

The meeting agenda is presented in Table A1. All sessions were held virtually.

**Table A1:** Symposium agenda.

Session	Speakers
Day 1: The value of early detection across multiple tumor types	Moderator: Dr. Monika Slovinec D’Angelo, Chief Research Officer, CCRAN
Symposium opening	Ms. Filomena Servidio-Italiano, M.A, President & CEO, CCRAN Mr. Allen Chankowsky, Metastatic Cancer Patient Advocate
Key Learnings from CCRAN’s 2021 and 2022 EAOCRC symposia	Dr. Michael Raphael, GI Medical Oncologist, Sunnybrook Health Sciences Centre, Toronto Dr. Clarence Wong, Gastroenterologist, University of Alberta
The global trend in early onset cancer	Dr. Tomotaka Ugai, Dana-Farber/Harvard Cancer Center, Cambridge MA
System-level challenges to accessing screening and timely diagnosis for early-onset cancers	Patient Group Roundtable Moderator: Ms. Martha Raymond, GI Cancers Alliance Inc. (US) Panel: Colorectal Cancer: Ms. Filomena Servidio-Italiano, CCRAN Lung Cancer: Ms. Michele Wright, Lung Cancer Canada Breast Cancer: Ms. MJ DeCoteau, Rethink Breast Cancer Cervical Cancer: Ms. Teresa Norris, Founder and President, HPV Global Action Ovarian Cancer: Ms. Elise Gasbarrino, Founder & Executive Director, Pink Pearl Canada Pancreatic Cancer: Ms. Stefanie Condon-Oldreive, President & CEO, Craig’s Cause Pancreatic Cancer Society
Identifying and addressing the psychosocial needs of younger cancer patients across the continuum of care	Panel Session Moderator: Ms. Marlie Smith, Clinical Nurse Specialist, AYA Oncology Program at Princess Margaret Hospital, Toronto, ON Presentation: Dr. Abha Gupta, Medical Director, AYA Oncology Program at Princess, Margaret Hospital, Toronto, ON Panel: Colorectal cancer journey: Ms. Jessica Dasler, Colon cancer patient Breast cancer journey: Ms. Alyson Geary, Breast cancer survivor and Lead, Impact Partnerships, Support & Community Powered Projects, Rethink Breast Cancer Lung cancer journey: Ms. Lindsay Hlushak, Lung cancer survivor Cervical cancer journey: Ms. Joanna Kirsh, Cervical cancer patient Cholangiocarcinoma journey: Ms. Teresa Holmes, Caregiver and Co-Founder, Hepatocellular Cholangiocarcinoma Canada
From detection to diagnosis, treatment and survivorship: tailoring care to the needs of young-onset cancer patients	Panel Session Moderator: Dr. Petra Wildgoose, Family Physician and Lead, Young Adult Colorectal, Cancer Program at Odette Cancer Centre, Sunnybrook Health Sciences Centre, Toronto, ON Medical oncologists: Colorectal cancer: Dr. Safiya Karim, Medical Oncologist and Clinical Assistant Professor, Tom Baker Cancer Centre, University of Calgary; Medical Director, Integrative Oncology Clinic Lung cancer: Dr. Shaqil Kassam, Medical Oncologist, Southlake Stronach Regional Cancer Centre, Newmarket, ON Breast cancer: Dr. Nancy Nixon, Medical Oncologist, Tom Baker Cancer Centre, Calgary, AB Gynecologic cancers: Dr. Sarah Hamilton, Radiation Oncologist, BC Cancer Agency, Vancouver, BC Pancreatic Cancers: Dr. Ravi Ramjeesingh, Medical Oncologist, Nova Scotia Cancer Centre & Dalhousie University, Halifax, NS Radiology Perspective: Dr. Ania Kielar, President, Canadian Association of Radiologists
How do we achieve timely diagnostic testing? what is the role of real-world evidence?	Panel Session Moderator: Mr. Don Husereau, BSc. Pharm MSc. Health Economist, Faculty of Medicine, University of Ottawa, ON Panel: Value-based healthcare (VBHC) Expert: Dr. Monika Slovinec D’Angelo, Ph.D. Health Scientist, VBHC Expert, Chief Research Officer at CCRAN Medical Oncologist: Dr. Ravi Ramjeesingh, Nova Scotia Cancer Centre & Dalhousie University, Halifax, NS Pathologist: Dr. Aaron Pollett, Anatomic Pathologist, Director of Division of Diagnostic Medical Genetics, Mount Sinai Hospital, Toronto, ON HTA perspective: Ms. Sylvie Bouchard, Director, Institut national d’excellence en santé et services sociaux (INESSS), Québec, QC Healthcare system perspective: Dr. Helen Anderson MBchB, FRACP, MD, FRCPC, Provincial Medical Director, Systemic Therapy at BC Cancer Centre, Provincial Health Services Authority, Vancouver, BC
Accessing clinical trials for the management of advanced disease across multiple tumor types	Moderator: Dr. Monika Slovinec D’Angelo Presentations: Dr. Dawn Richards, Ph.D, Director of Patient and Public Engagement, Clinical Trials Ontario Dr. Stephanie Michaud, Ph.D. President, BioCanRX Dr. Eric Chen, GI Medical Oncologist, Clinical Trials Investigator, Princess Margaret Cancer Centre, Toronto, ON Mr. Allen Chankowsky, Cancer patient advocate Mr. Jim Palma, Executive Director, TargetCancer Foundation, Cambridge, MA
Current and future research directions for early-onset cancers	Presentation: Dr. Shuji Ogino, Harvard T.H. Chan School of Public Health, Professor in the Department of Epidemiology; Professor of Pathology, Harvard Medical School, and Brigham and Women’s Hospital, Cambridge, MA
Day 2: Optimizing early age onset colorectal cancer care and outcomes	Moderator: Dr. Monika Slovinec D’Angelo, Chief Research Officer, CCRAN
Understanding the impact of a colorectal cancer diagnosis: Considering the patient journey across disease stages	Panel Session Moderator: Ms. Cassandra Macaulay, Senior Manager of Programs & Education, CCRAN Patient Panel: Stage II colon cancer survivor: Ms. Atoosa Rashid Stage III rectal cancer survivor: Ms. Armina Ligaya Stage IV colon cancer patient: Mr. Steve Slack Stage IV rectal cancer patient: Ms. Hayley Painter
Identifying and addressing the unique needs of younger colorectal cancer patients	Panel Session Moderator: Dr. Petra Wildgoose, Family Physician and Lead, Young Adult Colorectal Cancer Program at Odette Cancer Centre, Sunnybrook Health Sciences Centre, Toronto, ON Panel: Patient Expert: Dr. Marko Yurkovich, MD, Primary care physician and stage IV colorectal cancer patient Stage IV colon cancer patient: Ms. Suzanne Wood, Professional, young mother, metastatic cancer patient Radiation Oncologist: Dr. Rob Rutledge, MD, FRCPC, Radiation Oncologist at the Nova Scotia Cancer Centre, the QE II Health Centre, Halifax, NS Fertility Expert: Dr. David Gurau, Obstetrician and Reproductive Endocrinologist, Generation Fertility Patient Support Program Lead: Ms. Cassandra Macaulay, Senior Manager of Programs & Education, CCRAN
Advancements in colorectal cancer diagnostics and treatments: The role of Comprehensive Genomic Profiling (CGP)	International & Health Policy Presentations: Dr. José Perea, Colorectal Surgeon, Surgery Department, Jimenez Diaz Foundation University Hospital, Madrid, Spain Dr. Christopher Lieu, GI Medical Oncologist, Associate Director Clinical Research, Co-Director, GI Medical Oncology, University of Colorado, Aurora, CO Dr. Emina Emilia Torlakovic, MD, Ph.D. Head, Division of Hematopathology at SaskHealth, Founder and Director, Canadian Biomarker Quality Assurance (CBQA)
Preventing early age onset colorectal cancer through earlier screening programs for hereditary syndromes	Presentation: Dr. Kim Ma, Medical Oncologist, Segal Cancer Centre at Jewish General Hospital, Montreal, QC
Improving the diagnosis and management of hereditary colorectal cancer	Panel Session Moderator: Dr. Petra Wildgoose, Family Physician and Lead, Young Adult Colorectal Cancer Program at Odette Cancer Centre, Sunnybrook Health Sciences Centre, Toronto, ON Panel: Hereditary Cancer Patient Expert: Ms. Claudia Martino, CMMRD Syndrome Patient Medical Oncologist: Dr. Michael Raphael, GI Medical Oncologist, Sunnybrook Health Sciences Centre, Toronto, ON Surgical Oncologist: Dr. Usmaan Hameed, North York General Hospital, Toronto, ON Genetic Counsellor: Ms. Laura Palma, MSc, CCGC, CGC Certified Genetic Counsellor, Medical Genetics Assistant Professor, McGill University, Montreal, QC
The treatment of early-stage disease	Panel Session Moderator: Dr. Chris Hiller, Ph.D. Survivor of Stage II Rectal Cancer Panel: Patient Expert: Ms. Catherine Mason-Mifsud, Stage II Rectal Cancer Survivor Surgical Oncologist: Dr. Usmaan Hameed, North York General Hospital, Toronto, ON Medical Oncologist (US): Dr. Aparna Parikh, GI Oncologist, Director, Massachusetts General Hospital Cancer Center’s Global Cancer Care Program, Cambridge, MA GI Medical Oncologist: Dr. Andrea Cercek, GI Oncologist, Memorial Sloan Kettering Cancer Center, New York City, NY
The role of the microbiome in gastrointestinal cancers	Presentation & Panel Session Moderator: Dr. Sharlene Gill, GI Medical Oncologist, BC Cancer Agency, Vancouver, BC Panel: Dr. Pavlina Spiliopoulou, Medical Oncologist, Assistant Professor, University of Glasglow, UK Dr. Anna Spreafico, Head and Neck Medical Oncologist, Princess, Margaret Hospital and Site Lead and Clinician Investigator within the Tumor Immunotherapy Program, Toronto, ON Mr. Marcelino Dolores, Colorectal cancer patient expert
The treatment of advanced stage disease: improving patient outcomes through surgical resection	Presentations Dr. Marcelo Cypel, Thoracic Surgical Oncologist, University Health Network, Toronto, ON Dr. Gonzalo Sapisochin, Hepato-Pancreato-Biliary Surgical Oncologist, Toronto General Hospital, Toronto, ON Dr. Paul Karanicolas, Hepatobiliary Surgical Oncologist, Sunnybrook Health Sciences Centre, Toronto, ON Dr. Anand Govindarajan, Surgical Oncologist, Mount Sinai Hospital, Toronto, ON
Nutritional guidelines for supporting the patient’s cancer journey and symptom management	Presentation: Ms. Felicia Newell, RD, MSc, Registered Dietitian
Closing remarks	Ms. Filomena Servidio-Italiano, M.A., President & CEO, CCRAN

## Appendix B. Medical Advancements in Early-Age Onset Colorectal Cancer

Information was shared on the following medical advancements in early-age onset colorectal cancer (EAOCRC), with the intention of exploring the possibility of applying these new techniques to other disease sites.

### Appendix B.1. Precision Risk Stratification through Genomic Testing

Expert presenters agreed that all EAOCRC patients should be tested for germline and sporadic mutations. Two basic approaches were discussed in the context of assessing the patient’s level of risk and identifying potential targets for treatment.

The first approach—targeted sequencing of a handful of genes using smaller panels—identifies the majority of clinically actionable genetic mutations. Patients benefit by not having to wait several weeks for results before starting treatment. Moreover, surgical strategies could be modified according to results in some germline pathogenic mutations (e.g., in Lynch syndrome cases), which confer high risk of metachronous CRC development. The recently released Delphi Initiative for Early-Onset Colorectal Cancer (DIRECt) International Management Guidelines recommends germline genetic testing including a minimum of 14 specified biomarkers and, if feasible, a further 11 autosomal biomarkers and 8 genes [41].

At the other end of the spectrum, comprehensive genomic profiling (CGP) sequences 500–600 genes and can therefore identify mutations that are rare [42]. The National Comprehensive Cancer Network (NCCN) in the United States recommends genetic counselling and CGP for all patients with EAOCRC [43]. While CGP testing may be cost-prohibitive, its proponents argue that greater downstream treatment costs are avoided. The price of CGP is expected to decline in the future as mass testing drives economies of scale. Data gathered from CGP can be used to identify hereditary and environmental risk factors; to profile the cancer risk of individual patients and their families; and to aid researchers by correlating specific genomic alterations with disease features.

### Appendix B.2. Immunotherapy

Immunotherapies have revolutionized care for patients with CRC where the tumor harbors deficient mismatch repair proteins (dMMR) or is microsatellite instability high (MSI-H). Immunotherapy is already the first-line standard of care for patients with dMMR or MSI-H metastatic colorectal cancer [44], and recent studies have shown considerable promise in the setting of earlier stage CRC.

For example, the NICHE study identified that among 32 patients with dMMR colon cancer, a single dose of ipilimumab and two doses of nivolumab before surgery led to a 100% pathological response rate, including a 97% rate of major and complete pathological responses, respectively [45]. Similarly, among 12 patients with dMMR locally advanced rectal cancer (a tumor site of particular importance for younger patients) who were treated with neoadjuvant dostarlimab, 100% of patients achieved a complete clinical response and no patients required chemoradiation or surgery [46].

Thus, immunotherapies hold considerable promise to improve both quality of life and overall survival for patients affected by early-age onset colorectal cancers, and spare them from potential side-effects from chemotherapy, radiation and surgery. Intensive efforts are underway to try to identify ways to help patients with proficient mismatch repair protein (pMMR)/microsatellite-stable (MSS) colorectal cancer derive similar benefits from immunotherapy.

### Appendix B.3. The Microbiome

Symposium presenters summarized and discussed emerging research which strongly suggests a significant role of the gut microbiome in the etiology of EAOCRC [47,48]. The recent increased incidence in EAOCRC may be due, in part, to exposure to carcinogenic factors early in life, which has precipitated the generational shift towards a higher body mass index and obesity—a process mediated by the gut microbiome [49,50].

Additionally, a healthy gut microbiome has been shown to ameliorate chemotherapy toxicity and to improve host immunity and therapeutic response in cancer. Modulation of the microbiome—achieved through diet, chemotherapeutic intervention, or by fecal microbial transplantation—has become a novel strategy for prevention and treatment of CRC [51,52]. Targeting the microbiome also offers a way to increase the selection and boost the response of patients who might benefit from immunotherapy [53].

### Appendix B.4. Surgical Treatment of Early Stage and Advanced EAOCRC

Several advancements in the surgical treatment of early and advanced cancers were presented by expert panel members. These demonstrate a personalized approach that considers the unique needs of younger patients, including fertility preservation, minimization of permanent organ damage due to aggressive treatments, and the need to return to life priorities as quickly as possible.

In early-stage EAOCRC, minimally invasive approaches are now being considered for patients whose personal priorities align with clinical eligibility criteria. Laparoscopic surgery is now considered the standard-of-care for colorectal cancer.

For late-stage CRC, advancements in surgical techniques were discussed for treating metastases to the liver, peritoneum and lung. Younger patients may benefit from these developments because they tend to be diagnosed at a later stage of disease and to have higher levels of fitness to tolerate more aggressive therapy options.

Techniques that have shown promise for EAOCRC patients include:

- Chemotherapy infusion through the hepatic artery, which directly targets liver metastases and allows for the possibility of curative intent through surgical resection;
- Living donor liver transplantation for unresectable liver metastases [54,55];
- Hyperthermic intraperitoneal chemotherapy to enhance cytoreductive surgery for peritoneal metastases [56] (in future, some patients may qualify for immunotherapy as an alternative option to this technique);
- In vivo perfusion of high dose chemotherapy to the lung to target metastases directly.

### Appendix B.5. Decentralized Clinical Trials

Patient representatives at the symposium repeatedly raised concerns about the difficulty in accessing clinical trials in Canada. Once standard-of-care therapies for treating metastatic disease have been exhausted, patients seeking access to experimental therapies through clinical trials find that very few are available in Canada. Moreover, EAOC patients who live outside research centers are often unable to participate in studies due to travel costs and time away from their responsibilities.

The concept of decentralized clinical trials was discussed as an alternative methodology to encourage greater numbers of clinical trials to be initiated and to enable access by a geographically diverse population of patients. In this model, studies are designed to be conducted at locations convenient to participants, including their homes and local healthcare facilities. Digital technologies capture health information from individuals and their providers and enable direct communication with the research team.

For investigators, the decentralized model requires less time and expense and can greatly facilitate patient recruitment and retention. Data quality can be higher because collection is more frequent and information can be gathered about participants’ routine activities, providing insight into the effectiveness and safety of the treatment in “real life”. Services such as CGP and specialized lab testing still require centralization; however, the decentralized model offers the benefit of consistency of data analysis and interpretation.

A leading example of a decentralized approach is the Target Rare Cancer Knowledge (TRACK) trial, a patient- and advocacy-group-driven trial being conducted by TargetCancer Foundation in the United States. This observational study enrolls patients virtually from across the United States. Patients’ inclusion in the study directly impacts their care while gathering information about rare cancers. Patients enroll themselves using remote tools and undergo CGP testing. Results are interpreted by a molecular tumor board which makes recommendations for treatment to the patient and their treating physician. The centralized tissue repository facilitates future research into rare cancers.

### Appendix B.6. Healthcare System Adoption of Advancements

Timely and equitable access to advancements in EAO cancer care is crucial for patients living with these devastating diseases. Access by EAOC patients in Canada to newly approved technologies is fragmented across jurisdictions and, in some instances, is dependent on the patient’s ability to pay. This situation threatens to worsen as personalized medicine continues to evolve and each institution acts independently to adopt the new technologies.

Encouragingly, there is increasing recognition of the value that patients can contribute to integrated approaches to policy-making. Patients play valuable roles in defining meaningful outcomes, raising awareness of equity issues for remote and rural patients, and by affirming patients’ priorities. Symposium presenters from organizations across Canada shared their approaches to policy-making and to establishing national quality standards and adoption guidelines that will help to speed access to new technologies for EAOC patients.
